# A Channel Rendezvous Algorithm for Multi-Unmanned Aerial Vehicle Networks Based on Average Consensus

**DOI:** 10.3390/s23198076

**Published:** 2023-09-25

**Authors:** Yunlu Wang, Bo Zhang, Shan Qin, Jinlin Peng

**Affiliations:** National Defense Innovation Institute, Fengtai District, No. 53 East Street Courtyard, Beijing 100071, China; yunlu_wang@163.com (Y.W.);

**Keywords:** channel rendezvous, distributed multi-UAV networks, average consensus, adaptive network construction

## Abstract

Realizing the distributed adaptive network construction of multi-UAV networks is an urgent challenge, as they lack a reliable common control channel and can only maintain a limited sensing range in crowded electromagnetic environments. Multi-unmanned aerial vehicle (UAV) networks are gaining popularity in many fields. In order to address these issues, this paper proposes a multi-UAV network channel rendezvous algorithm based on average consistency. The goal of the algorithm is to adjust the communication channels of each UAV to converge on the same channel, since the communication link of the multi-UAV network is broken due to interference. The proposed memory-based average consistency (MAC) algorithm utilizes the network adjacency matrix as prior information. Furthermore, for the case where the adjacency matrix is unknown, this paper also proposes the Multi-Radio Average Consensus (MRAC) algorithm, which achieves a beneficial trade-off between rendezvous performance and hardware cost. Simulation results demonstrate that the proposed MAC and MRAC algorithms provide superior network convergence time and scalability in networks of different densities. Finally, a hardware simulation platform based on a multi-UAV network was designed using a software-defined radio platform, and experimental simulations were performed to prove the effectiveness of the MAC algorithm in a real environment.

## 1. Introduction

With the continuous development of science and technology, unmanned aerial vehicles (UAVs) are playing an increasingly important role in many fields. However, UAVs can only maintain a limited sensing range in complex electromagnetic environments and lack reliable public control channels. Therefore, realizing the distributed adaptive network construction of a multi-UAV network becomes an urgent challenge. Channel convergence is a kind of algorithm to realize the construction of UAV adaptive network, which aims to adjust the communication channels of each UAV to converge on the same channel [1,2]. Multi-UAV networks, on the other hand, can extend their durability [3], robustness [4], and flexibility to a larger range of application scenarios by combining numerous UAVs [5].

Maintaining communication is the foundation for multi-UAV networks to collaborate in order to complete their tasks. During operation, multi-UAV networks may be heavily interfered, and some representative cases are as follows:

- A multi-UAV network operates at unlicensed band, e.g., ISM band;
- A multi-UAV network operates as secondary users and a primary users presents [6];
- A multi-UAV network is intentionally jammed by adversarial emitters.

A simple solution to the aforementioned issues is to pre-program all UAVs with a backup channel for channel rendezvous. However, interference to the UAVs could also disturb the backup channel. In this case, network self-heading via channel rendezvous [7] becomes a viable solution.

Blind rendezvous algorithms [8,9] and reinforcement learning algorithms [10] are currently the two main methods for channel rendezvous. The blind rendezvous technique is primarily achieved through the frequency hopping algorithm, which generates a hopping sequence that allows two UAVs to simultaneously jump to the same channel and complete the rendezvous. On the other hand, methods that enable disconnected UAV pairs to synchronize their frequencies involve single-radio based approaches [11], clock-based ideas [12], and matrix-based frequency hopping sequences. However, these algorithms are time-consuming, as they require sequential restoration of communication links between UAVs. In contrast, reinforcement-learning-based channel rendezvous algorithms aim to minimize the collision probabilities between primary and secondary users [13], as well as reduce the expected rendezvous time by simultaneously adjusting the frequencies of all UAVs. Nevertheless, the learning process of such algorithms is time-consuming and becomes exponentially longer as the number of nodes increases.

The average consensus algorithm is a completely distributed algorithm for multi-UAV networks. It allows network nodes to average a specific variable by exchanging information only with their immediate neighbors. Therefore, if the variable is the channel index or frequency of the UAVs in the network, the average consensus algorithm can facilitate a new class of scalable and distributed channel rendezvous schemes. This paper is the first to design and compare different channel rendezvous schemes based on average consensus, as far as we know.

The optimal symmetric weights algorithm proposed by Lin Xiao and Stephen Boyd [14] is the classic average consensus algorithm, which maximizes the algorithm’s efficiency by configuring the connected edge weights. Furthermore, the convergence of algorithm is accelerated by using two-hop paths [15], stored memory [16], prediction functions [17], buffer-aided relays [18], or by including non-linear terms [19]. In addition, to adapt to practical scenarios, further resource savings can be achieved by setting iteration threshold parameters [20,21] and dividing the subnetwork [22], using information compensation to solve the communication delay problem [23], using system noise to solve the node failure problem [24], and using subgradients to solve the asynchronous network problem [25]. The average consensus algorithm is used to implement the multi-UAV network channel rendezvous algorithms proposed in this paper. The algorithms have the advantage of fast convergence in a fully distributed manner.

In this paper, we have designed two efficient channel rendezvous algorithm for a large number of multi-UAV networks. We propose and compare two channel rendezvous algorithms based on average consensus. We consider a scenario in which the multi-UAV becomes disconnected due to interference during a mission and requires a channel rendezvous to re-establish network-wise connection.

The contributions of this paper are as follows:

- We propose a novel Memory-based Average Consensus (MAC) algorithm. To the best of our knowledge, it is the first average-consensus-based channel rendezvous algorithm. It requires less rendezvous time than other state-of-the-art algorithms. For example, it requires only 164 time slots to complete channel rendezvous in a 100-UAV network;
- In the absence of the adjacency matrix, we propose a Multi-Radio Average Consensus (MRAC) algorithm. It is shown that by tuning the multi-radio configuration, the MRAC may achieve a beneficial tradeoff between rendezvous performance and hardware cost. When each UAV has two radios, for example, the channel rendezvous in a 100-UAV network requires 104 time slots to be completed;
- Hardware implementations of MAC algorithm are conducted on a software-defined radio platform based on the Zynq7045 and AD9361 chipsets. The results validate the feasibility of MAC in practical system, and it is also pinpointed that the frequency sensing error should be minimized to ensure convergence performance.

The rest of the paper is organized as follows. The network model and problem formulation are described in Section 2. The channel rendezvous algorithms based on average consensus are provided in Section 3. The validity of the MAC algorithm’s hypothesis is examined in Section 4. Additionally, a performance analysis is carried out and the proposed algorithms are compared with state-of-the-art algorithms. In Section 5, physical experiments are carried out in conjunction with self-developed software-defined radio hardware. The effects of spectrum sensing errors and the number of iterations on rendezvous success ratio is also analyzed in this section. Finally, the conclusion is provided in Section 6.

## 2. System Model and Problem Formulation

### 2.1. System Model

We consider a scenario like this: the communication frequency of a multi-UAV network is interfered and cannot support quality of service. The pre-defined alternate channel is also unavailable due to interference. Therefore, the entire UAV network needs to find and converge to another non-interfered channel in the channel set. The involved system parameters are shown in Table 1.

In this paper, we consider a multi-UAV network that operates in $N_{c}$ non-overlapping frequency bands. Each band represents a channel. As a result, the channel set can be expressed as follows:

(1) $$C=\left\{c_{1},c_{2},\cdots ,\right.\left.c_{N_{c}}\right\}$$

The multi-UAV network is made up of *m* UAVs. Each UAV is outfitted with a half-duplex radio for spectrum sensing and broadcasting information. In this paper, we assume that the radio can detect any frequency in the available frequency band and broadcast data at any frequency in the available frequency band. All UAVs have the same range for spectrum sensing. The spectrum sensing range of each UAV is represented by a sphere with itself as the center and $R_{s}$ as the radius. A communication link can be formed between two UAVs with three-way handshake if they are within each other’s spectrum sense range.

The adjacency matrix of the multi-UAV network is denoted by A and can be expressed as:

(2) $$A=\left[\begin{array}{cccc} A_{11} & A_{12} & \cdots & A_{1m}\\ A_{21} & A_{22} & \cdots & A_{2m}\\ \vdots & \vdots & \ddots & \vdots \\ A_{m1} & A_{m2} & \cdots & A_{mm} \end{array}\right]$$

We set the distance between UAV *i* and UAV *j* as $D_{ij}$, $A_{ij}$ is defined as follows:

(3) $$A_{ij}=\left\{\begin{array}{cc} 0 & i=j\;\mathrm{or}\;D_{ij}>R_{s}-\Delta r\\ 1 & i\neq j\;\mathrm{and}\;D_{ij}\leq R_{s}-\Delta r \end{array}\right.$$

where Δr indicates the buffer distance; the purpose of setting it will be explained later. The adjacency matrix A is stored in each UAV’s memory, and is updated in real time as the network topology changes.

The neighboring UAV of UAV *i* is named as $Neigh_{i}$, written as follows:

(4) $$Neigh_{i}=\left\{j|A_{ij}=1,\;j=1,2,\ldots ,m\right\}$$

### 2.2. Problem Formulation

This paper aims at minimizing rendezvous time and solves the following problem based on satisfying the above model.

After the communication link is disconnected, the frequency of UAV *a* at time slot *t* is $f_{a}\left(t\right)$. Thus, the frequencies of all UAVs in the entire multi-UAV network are represented as vectors $f\left(t\right)={\left[f_{1}\left(t\right),f_{2}\left(t\right),\cdots ,f_{m}\left(t\right)\right]}^{T}$. Our goal is to have all of the UAVs jump to the same channel, further completing the channel rendezvous.

To compare the performance of different algorithms, we use the Time-To-Rendezvous (TTR) as a performance metric. TTR is the number of time slots consumed by all UAVs in a multi-UAV network between the time the communication link is disconnected and when it is fully established. The preceding issue is formalized as follows:

(5) $$\begin{array}{cc} \min & \;TTR\;\\ \;s.t.\; & \forall 1\leq i,j\leq m,i\neq j,f_{i}\left(TTR\right)=f_{j}\left(TTR\right). \end{array}$$

The primary goal of this paper is to create a high-performance channel rendezvous algorithm for a multi-UAV network in order to make all UAVs quickly converge to the same channel and establish network-wise connections.

## 3. Algorithm Design

In this section, we propose two average consensus-based channel rendezvous algorithms: the Memory-based Average Consensus algorithm (MAC) [16], and the Multi-Radio Average Consensus algorithm (MRAC). Both algorithms only require the UAV to sense the spectrum information of neighboring UAVs.

### 3.1. Memory-Based Average Consensus (MAC) Algorithm

The MAC algorithm achieves channel rendezvous by sensing and memorizing neighboring UAVs’ frequency information. For UAV *i*, i=1,2,⋯,m, it can obtain frequency information about other UAVs by sensing channel frequency and resolve the ID of its neighboring UAVs via radio. In each iteration of the MAC algorithm, each UAV implements a single round of spectrum sensing. The iteration stops when all UAVs are able to calculate the average frequency. Further, all UAVs converge to the channel that the average frequency belongs to and complete channel rendezvous by three-way handshake. More details of the algorithm are shown in Algorithm 1.

The adjacency matrix kept in the UAV memory determines which UAVs are broadcast in what order throughout each iteration. We assume that no neighbors of UAV will exceed its maximum effective communication range from the moment that all UAVs break the communication link until the channel rendezvous is complete. Therefore, we introduce a buffer distance Δr based on the UAV’s maximum velocity.

**Figure 1:**
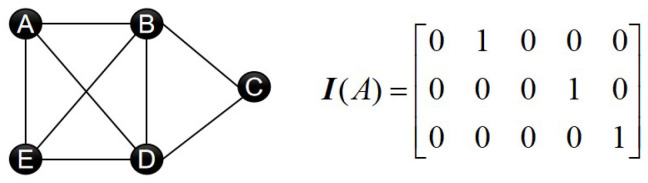
A 5-node network for describing the matrix I(i) and the fully coupled subnets: the matrix I(A) of node *A* is illustrated on the right. Except for subnets ABDE and BCD, any two connected nodes and their connected edges can form a fully coupled subnet. The network is only used to illustrate the fully coupled sub-network, and the UAV network used in the simulation is generated at random.

After disconnecting the multi-UAV network from the communication link, for UAV *i*, run Algorithm 1 with the network adjacency matrix in memory and the UAV’s current frequency $f_{i}\left(0\right)$ as input. Lines 1∼6 are the data pre-processing part of the algorithm. I(i) is a matrix made up of the rows of the identity matrix whose indexes are included in UAV *i*’s neighbors. For example, Figure 1 shows a five-node network and the matrix I(A) which the node A correspond to. $s_{i}\left(t\right)$ denotes the data saved at UAV *i* after *t* iterations. At each iteration, UAV *i* saves the average frequencies of the neighboring UAVs. In particular, $s_{0}$ is the initial frequency of UAV *i*, $f_{i}\left(0\right)$. $s_{i}\left(t\right)$ can be expressed as:

(6) $$s_{i}\left(t\right)=F_{i}{\left(t\right)}^{T}f_{i}\left(0\right)=\left(\begin{array}{c} {e_{i}}^{T}\\ I(i)(M{A)}^{0}\\ I(i)MA\\ \vdots \\ I(i)(M{A)}^{t-1} \end{array}\right)f_{i}\left(0\right)$$

**Algorithm 1** Memory-based Average Consensus algorithm**Input:** A, $f_{i}\left(0\right)$, *m***Output:** $\bar{f}$
  1:1← The vector with all coefficients ones;  2:M← Diagonal matrix consisting of the reciprocal of the sum of the elements of each row of A;  3:I(i)← the matrix composed of the rows of the identity matrix whose indexes are included in the neighbors of UAV *i*;  4:$s_{i}\left(t\right)\leftarrow$ Data saved in the UAV *i* after *t* iterations;  5:$F_{i}\left(t\right)\leftarrow$ Coefficient of data saved in the UAV *i* after *t* iterations;  6:**for** int *j* = 1 to *m* **do**  7:    t=0;  8:    **while** $rank\left(F_{j}\left(t\right)\right)\neq rank\left(F_{j}\left(t\right)|e\right)$ **do**  9:          t=t+1;  10:        $F_{j}\left(t\right)=\left[F_{j}\left(t-1\right),I(M{A)}^{t-1}\right]$;  11:    **end while**  12:    r(j)=t;  13:
**end for**
  14:$R=\max_{j}\;\;r\left(j\right)$;  15:subnet=SUBNETTING(A);  16:$N_{s}\leftarrow$ Number of subnet;  17:**for** int *t* = 0 to *R* **do**  18:    **for** int *k* = 1 to $N_{s}$ **do**  19:        **if** UAV *i* is belongs to subnet(k) **then**  20:           **if** *t* == 0 **then**  21:               Broadcast $f_{i}\left(0\right)$;  22:           **else**  23:               ${\bar{f}}_{i}\left(t-1\right)=\sum_{j\in Neigh_{i}}f_{j}\left(t-1\right)$;  24:               Broadcast ${\bar{f}}_{i}\left(t-1\right)$;  25:           **end if**  26:        **else**  27:           Sensing and saving the frequency information  28:        **end if**  29:    **end for**  30:
**end for**
  31:$w_{i}=\frac{1}{m}F_{i}{\left(r(i)\right)}^{T}{\left(F_{i}\left(r\left(i\right)\right)F_{i}{\left(r(i)\right)}^{T}\right)}^{-1}1$;  32:$\bar{f}=s_{i}{\left(r(i)\right)}^{T}w_{i}$;  33:
**return** $\bar{f}$




Lines 7∼14 compute the minimum number of rounds R of the required iteration. r(j) denotes the smallest number of rounds required for UAV *j* to compute the average frequency, implying that *w* exists such that (7) has a solution.

(7) $$s_{i}{\left(t\right)}^{T}w_{i}=\bar{f}$$

Equation (7) can also be simplified to (8):

(8) $$F_{i}\left(t\right)w_{i}=\frac{1}{m}1$$

Equation (8) has a solution under the condition $rank\left(F_{i}\left(t\right)\right)=rank\left(F_{i}\left(t\right)|1\right)$. *R* is the minimum number of iterations for which the average frequency can be calculated for any UAV.

The next step is for each UAV to collect information from neighboring UAVs. However, if the UAV broadcasts the information sequentially and the other UAVs sense it, only the broadcasting UAV and its neighboring UAVs are involved in each broadcast. Nevertheless, this approach is deemed ineffective.

To increase time slot utilization and optimize iterations, we employed subnetting techniques to divide the network. Each subnet, represented by a circle, consists of multiple time slots. Within each time slot, multiple UAVs in the same subnet broadcast simultaneously, while UAVs in different subnets sense automatically. To prevent simultaneous broadcasts among neighboring UAVs and ensure information exchange, we introduced the concept of subnet division. This means that neighboring UAVs cannot be placed in the same subnet. Algorithm 2 describes the precise process of subnet division. The method involves multiple cycles, each exclusively handling unsubnetted UAVs. We initialize a new subnet and iterate through the UAVs in a single loop. A UAV is allocated to the new subnet only if none of its neighboring UAVs are already included in it.

Lines 18∼30 depict the iterative process. The weight vector $w_{i}$ in (7) can be calculated at the end of the iteration using the formula shown in line 31. Eventually, the average frequency can be calculated in line 32.
**Algorithm 2** Subnetting function**Input:** A**Output:** all subnets  1:**function** subnetting(A)  2:    i=0;  3:    **while** Presence of UAVs still not subnetted **do**  4:        subnet(i)={};  5:        **for** UAV *j* is not subnetted **do**  6:           **if** $Neigh_{j}\cap subnet\left(i\right)=\emptyset$ **then**  7:               subnet(i)=subnet(i)∪j;  8:           **end if**  9:        **end for**  10:      i=i+1;  11:    **end while**  12:    **return** all subnets  13:**end function**

### 3.2. Multi-Radio Average Consensus (MRAC) Algorithm

In order to address the channel rendezvous problem in multi-UAV networks, the MAC algorithm is typically used and it is known to be effective according to Section 4. However, in practical situations, the UAV may face challenges that prevent it from obtaining the adjacency matrix of the network. For example, the UAVs might be vulnerable to destructive attacks or may fly too fast to remain within the buffer zone. To tackle this issue, we propose the MRAC algorithm, which is derived from the Floods algorithm. The MRAC algorithm is designed to enable channel rendezvous in multi-UAV networks under such circumstances.

The Flooding algorithm concept [26] is as follows: In the first iteration, all nodes send their own node values in vector form to their neighbors and receive node values from their neighbors in vector form, which are stored in memory. In the next iteration, the nodes send their previously unsent node values and receive the new node values sent by the neighboring nodes. If the received node values are previously unsent node values, they are stored in memory. A node will not stop broadcasting until the values of all nodes have been broadcast. If the network is connected, any node can obtain the node values of all nodes after several iterations. Thus, the average node value can be calculated.

The Flood algorithm cannot be directly used in a multi-UAV network because the UAV can only broadcast on one channel. The implementation of the Flood algorithm requires all UAVs to be equipped with *m* radios, which is not practical in multi-UAV networks.

Considering the realistic constraints, to match the Flooding algorithm concept, we assume that each UAV is equipped with n (n≥2) radios. One of the radios is used to sense frequencies and resolve the ID of neighbors of UAV. The others are used to broadcast information. That is, there are n−1 radio frequency channels for each UAV. Each UAV can sense channel frequency and broadcast information at the same time. Under the above hypotheses, we propose the MRAC algorithm. It is more adapted to realistic conditions than the Flood algorithm. The detailed steps of the MRAC algorithm are shown in Algorithm 3.

Algorithm 3 begins by initializing the parameter S. S denotes the frequency data stored in node *i*. Lines 2∼5 depict the iterative process, in which each UAV broadcasts its own unbroadcast frequency information in a time slot, and listens to neighboring UAVs at the same time.

The MRAC algorithm does not require an adjacency matrix as priori information, but it requires a higher level of hardware capability to support efficient sensing. It requires each UAV to be equipped with multiple radios. Furthermore, the average efficiency of the two algorithms differs for multi-UAV networks with varying numbers of UAVs, which will be analyzed in Section 4. The next section may be divided by subheadings. It should provide a concise and precise description of the experimental results, their interpretation, as well as the experimental conclusions that can be drawn.
**Algorithm 3** Multi-Radio Average Consensus algorithm**Input:** ${f_{i}}^{0}$, $N_{c}$, *m***Output:** $\bar{f}$  1:S← Information of the frequency of each UAV stored in UAV *i*  2:**while** Presence of unbroadcasted UAV frequency information **do**  3:    Broadcast information about the first n−1 acquired but previously unbroadcasted UAV frequencies  4:    Sense the frequency of neighbor UAVs and storing the information in S  5:**end while**  6:$\bar{f}\leftarrow$ Average of all elements in S  7:**return **$\bar{f}$

## 4. Numerical Results

This section presents the simulation results for different sizes of multi-UAV networks. To simulate various scenarios, we randomly generate 100 UAV-connected networks with nodes of 10, 50, 100, 250, and 500, respectively. First, we examine if the MAC algorithm theory was acceptable. Following that, we evaluate the MAC and MRAC algorithms against other channel rendezvous algorithms. Finally, we examine the elements that affect efficiency as well as the scalability of the MAC algorithm and MRAC algorithm. The simulation platform is MATLAB 2016A, which is powered by an AMD Ryzen 7 5800H CPU.

### 4.1. Effect of Network Dynamics

In this section, we add a justification for the hypothesis required to complete the MAC algorithm. The hypothesis is described as from the time all UAVs are disconnected from the communication link to the completion of the channel intersection, the neighbors of any UAV do not fly out of their maximum effective communication range.

**Figure 2:**
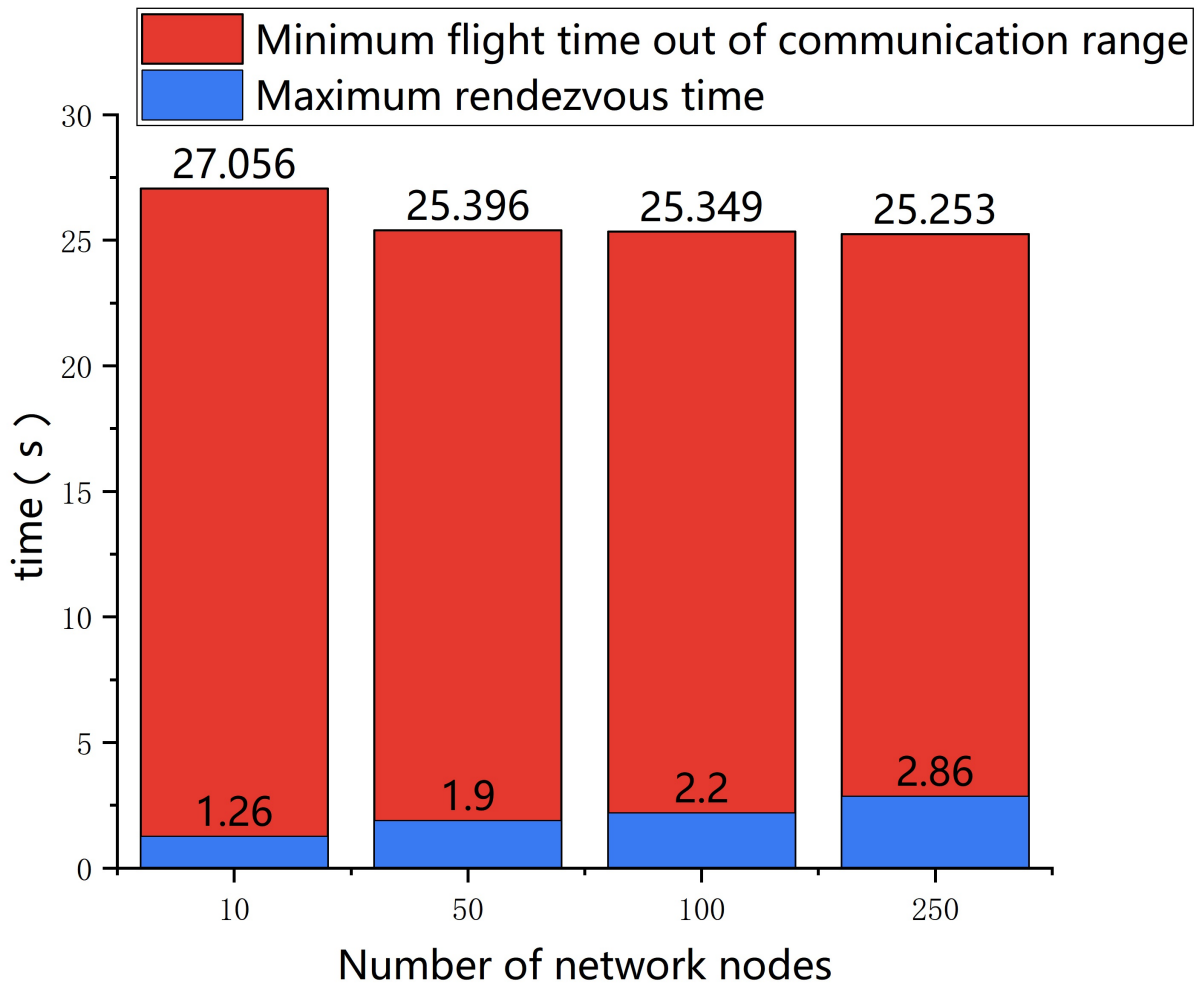
Comparison of the minimum time when a UAV’s neighbor exceeds its maximum effective communication range and the maximum time for the network to complete the channel rendezvous.

We verify the validity of the hypothesis through simulation experiments. In the simulation, we set *R* = 5 km and Δr = 1 km, and each time slot is equal to 10 ms according to IEEE802.22 [27]. Assume that the UAV travels at 20 m/s and continues to fly in the original direction after the UAV disconnects from the communication link. We record the time taken from the start of the disconnection until the neighboring UAV with the UAV flies out of its maximum effective communication range. The results are in Figure 2:

It can be seen that no UAV network with any number of nodes will have a minimum time when a neighboring UAV with UAV flies out of its maximum effective communication range of less than 25 s. However, this time is all much longer than the time required for the network to complete the channel rendezvous. Therefore, this hypothesis is reasonable.

### 4.2. Performance Evaluation

ATTR is the average TTR required by an algorithm to process a set of networks. It is a direct reflection of the efficiency of this algorithm in solving the channel rendezvous problem for multi-UAV networks. MTTR is the maximum TTR required by an algorithm to process a set of networks. MTTR reflects not only the rendezvous efficiency of the algorithm but also the algorithm’s stability when dealing with different networks.

In order to illustrate the performance of the MAC and MRAC algorithms, we compare both algorithms to three recently proposed blind rendezvous frequency hopping algorithms, namely the SJRW algorithm [12], the ABIO algorithm [8], and the PMTP algorithm [28]. The ABIO, SJRW, and PMTP algorithms are chosen because they have great performance when the number of channels is high, when dealing with the 2-user model, and in terms of energy consumption, respectively. In addition, we included the Flood algorithm in the comparison as an upper bound on the performance of the MRAC and MAC algorithms.

**Figure 3:**
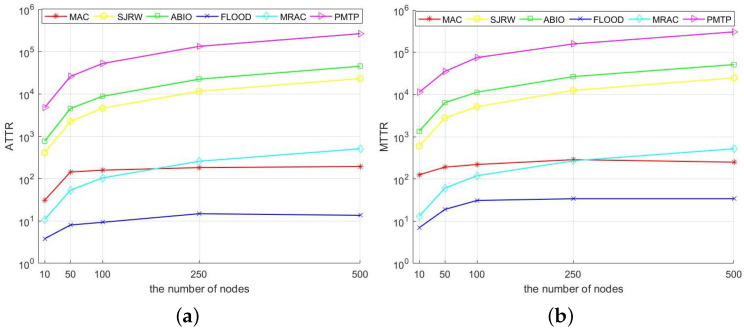
ATTR (**a**) and MTTR (**b**) required for each algorithm. The horizontal coordinate is the number of UAVs in the UAV network. TTR is the total number of time slots from the time that the multi-UAV network disconnects the communication link to the time that it completes reconnection. ATTR is the average TTR of the 100 randomly generated networks, and MTTR is the maximum TTR of the 100 randomly generated networks. MAC algorithm and MRAC algorithm (two radios) are the algorithms proposed in this paper. the SJRW algorithm [12], the ABIO algorithm [8], and the PTMP algorithm [28] are the algorithms to be compared. The FLOOD algorithm is difficult to be implemented in practical situations because it requires all UAVs to be equipped with multi-radio whose number is equal to the number of UAVs in the network. Therefore, the FLOOD algorithm is used as a benchmark of optimal convergence performance.

The simulation results are shown in Figure 3. Figure 3 depicts the ATTR and MTTR required by the five algorithms for a multi-UAV network with a various number of nodes. Because of the wide range of data results, logarithmic coordinates are used as horizontal coordinates. According to Figure 3, for the MAC algorithm, the ATTR and MTTR do not increase significantly with the number of nodes in the network after 50, indicating the algorithm’s superiority in handling the channel rendezvous problem in multi-UAV networks. It consistently outperforms the SJRW algorithm, the ABIO algorithm, and the PMTP algorithm. The MRAC algorithm also outperforms the SJRW, ABIO, and PMTP algorithms in networks of different sizes. It performs better than the MAC algorithm until the number of nodes reaches 250. We use the aforementioned algorithm to perform channel rendezvous after the multi-UAV network was disconnected by interference. We set up 30 optional channels for the frequency hopping algorithms ABIO, SJRW, and PMTP. Considering the load limits of the UAV, we outfitted each UAV with two radios for the MRAC algorithm.

It is important to note that Figure 3 demonstrates the MAC algorithm’s superiority when dealing with networks that contain various amounts of nodes. On the other hand, the MRAC algorithm exhibits superiority and stability when dealing with small-scale networks.

### 4.3. Factors Influencing the Efficiency of the MAC Algorithm

The number of time slots required by the MAC algorithm is determined by two parameters: the number of subnets divided and the number of algorithm iterations. Both parameters are determined by the adjacency matrix of the network. In other words, when the network’s adjacency matrix is determined, the number of time slots required by the MAC algorithm is already determined. The total number of time slots equals the product of the number of subnets divided by the number of algorithm iterations.

#### 4.3.1. The Number of Subnets

The number of subnets depends on the network’s maximum fully coupled subnet. It is divided into a number of time slots in one iteration of the algorithm, during which all UAVs in one subnet broadcast messages, and all UAVs in the other subnets listen by default. As a result, the more subnets that are divided, the more time slots are required in a single iteration. It is known that any two nodes that are neighbors of each other cannot be divided into the same subnet due to the way the subnets are divided. If there are multiple nodes in the network that are neighbors of each other, i.e., these nodes can form a fully coupled subnet, then when the network is divided into subnets, these nodes all belong to different subnets. As a result, we can conclude that the number of subnets is equal to the number of nodes in the network’s maximum fully coupled subnet. For example, consider a network with five nodes, as shown in Figure 2. The largest fully coupled subnet in the network is subnet ABDE. Thus, the network can be divided into four subnets, with the final subnet number of each node being 1, 2, 1, 3, 4, respectively.

#### 4.3.2. The Number of Iterations of the Algorithm

This subsection will analyze the effect of the topological properties on the number of iterations. The number of algorithm iterations is dependent on the adjacency matrix of the network. In order to analyze the factors influencing the number of iterations of the algorithm, we compared some topological properties of the network with the number of iterations. These include:

- Diameter: The maximum number of hops between any two nodes in the network;
- Average degree: The average of all node degrees in the network, which is the important parameter to reflect the density of the network;
- Assortativity: The degree correlation of the network, with larger values indicating that nodes tend to be connected to nodes with larger degree values and smaller values indicating that nodes tend to be connected to nodes with smaller degree values.

**Figure 4:**
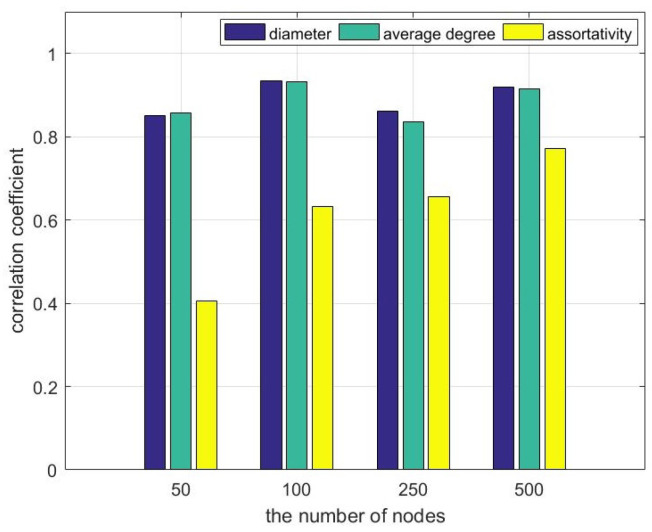
Correlation coefficients between various network topology properties and the number of iterations of the MAC algorithm.

To illustrate the relationship between the three topological properties of networks and the number of iterations, we generated 100 networks with 50, 100, 250, and 500 nodes, respectively, and calculated their diameters, average degrees, and assortativity coefficients. We run the MAC algorithm on the above networks. Then, we calculate the correlation coefficients between the number of iterations of the algorithm and the three topological properties of networks, as shown in Figure 4.

The correlation coefficients of network diameter and average degree with the number of iterations are greater than 0.8 for all sizes of networks, according to Figure 4. This indicates a strong positive correlation between the diameter of the network and the average degree of the network with the number of iterations. On the other hand, the correlation coefficients of network assortativity with the number of iterations increased with the number of nodes, but did not exceed 0.8 at the maximum. Therefore, the network assortativity does not correlate significantly with the number of iterations of the network. As a result, it is advisable to aim for smaller network diameters and network averages when designing network topology.

### 4.4. Factors Influencing the Efficiency of the MRAC Algorithm

**Figure 5:**
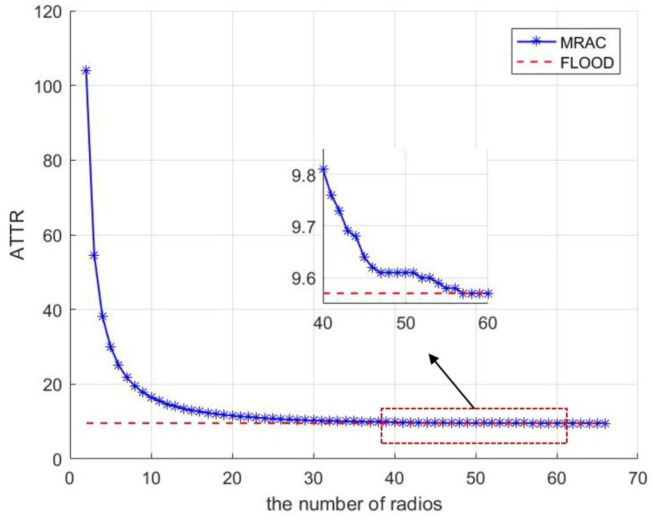
Relationship between the number of nodes and ATTR.

To explore the specific relationship between the number of radios and MRAC efficiency, we randomly generated 100 connected networks of 100 nodes. We conducted simulation experiments in the above network. The simulation result is shown in Figure 5. From the analysis in Section 3, we know that the main factor affecting the efficiency of the MRAC algorithm is the number of radios carried on the UAV. When the number of radios is small, the ATTR of the MRAC algorithm is dramatically reduced as the number of radios increases. After the number of radios reaches 10, the reduction in ATTR for each additional radio is less than 1. When the number of radios is increased from 2 to 10, the ATTR required by the algorithm is reduced by 87.65. The larger the radio number is, the less the algorithm performance improves. When the number of radios reaches 57, the MRAC algorithm reaches its best performance and converges to the same level as the Flood algorithm.

In summary, taking performance and cost into account, we choose the MRAC algorithm in such a way that we only need the MRAC algorithm with proper radio numbers to ensure good performance. For example, in a network of 100 UAVs, equipping each UAV with 3 and 4 radios may reduce 45.6% and 63.3% convergence time, respectively. Meanwhile, deploying 20 radios on a UAV may achieve an 89.1% reduction in convergence time. Therefore, selecting a reasonable number of radios may exploit the most benefits and control the hardware costs.

### 4.5. Effects of the UAV Network Density

**Figure 6:**
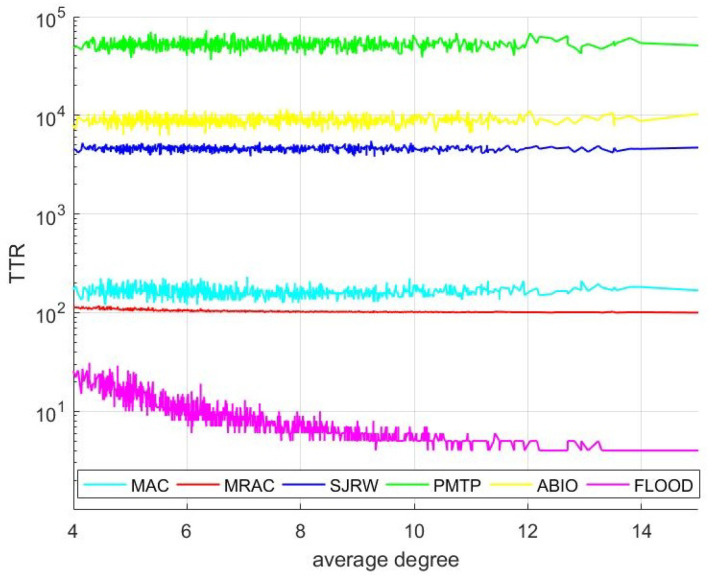
Relationship between the average degree and the Time-To-Rendezvous (TTR) of each algorithm.

To measure the network density, the average network degree serves as a significant parameter. The average network degree is determined by considering the number of connected edges as the degree of a node. In order to examine the efficiency of each algorithm with respect to the network’s average degree, we randomly create 1000 connected networks, each comprising 100 nodes. Subsequently, we implement each algorithm on these networks, and Figure 6 demonstrates the simulation results.

Figure 6 demonstrates the scalability and stability of the MAC algorithm and the MRAC algorithm. It is observed that there is no direct linear relationship between the performance of each algorithm and the network’s average degree. As the average degree of the network increases, the TTR required to complete channel rendezvous for the SJRW, ABIO, PMTP, and MAC algorithms experiences small fluctuations. However, the MRAC algorithm exhibits stable and good performance even when the average degree of the network exceeds 10. This is because the TTR required by the frequency hopping algorithms mentioned earlier is primarily influenced by the number of nodes in the network, rather than the network’s average degree. It is worth noting that the TTR of the MRAC algorithm is associated with the number of radios, rather than the average degree.

## 5. Hardware-in-the-Loop System Setup

**Figure 7:**
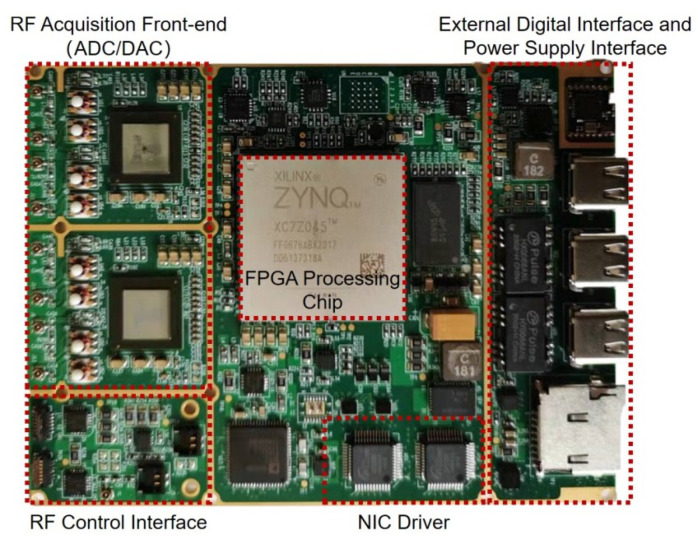
Self-developed sensing and communication integration module. The module dimensions are 103 mm × 79 mm. The total weight is 78 g. In this module, RF Acquisition Front-end is used to sense the frequency signals of other modules. The RF control interface is used to adjust the signal broadcast by the module.

In order to further validate the MAC algorithm, physical validation has been added to the numerical simulation. This is necessary because accurately modeling errors in the practical environment is challenging. The effectiveness of the MAC algorithm is verified through physical experiments conducted with the relevant hardware. The hardware used is a self-developed sensing and communication integration module. It facilitates the regulation of the broadcast frequency and sensing of other modules’ broadcast frequency within a certain range, enabling full satisfaction of the MAC algorithm’s hypotheses. Figure 7 shows a photograph of the module used in this experiment.

### 5.1. Setup of the Physical Experiment

**Figure 8:**
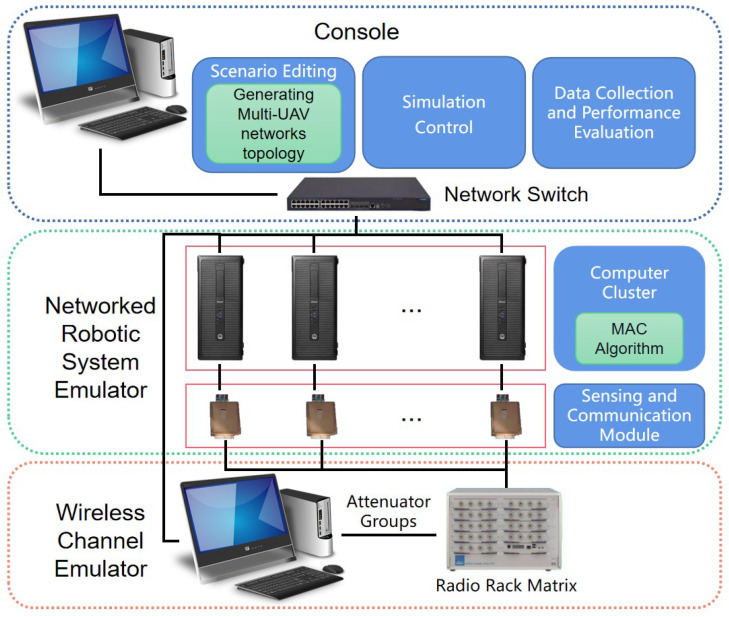
The hardware-in-the-loop (HIL) architecture of multi-UAV network simulation system.

In order to verify the effectiveness of the MAC algorithm, physical experiments were conducted in combination with commercial software-defined radio hardware. The system architecture of this physical experiment is depicted in Figure 8 [29]. The experimental system consists of three components: the console system, the networked robotic system simulator, and the wireless channel simulator. The console system allows for scene editing, simulation control, simulation data collection, and performance evaluation. During scenario editing, a randomly connected network topology is generated, which includes the coordinate information of each node as well as the network’s adjacency matrix. The simulation control is used to execute the behavior of multi-UAV networks and communication terminals. In the simulation control, a random set of initial frequencies of simulated UAVs is generated and the serial number of each simulated UAV is defined. The connected robotic system simulator is composed of a computer cluster and six sensing and communication modules. Each group consisting of a computer and a module simulates a UAV in the distributed Multi-UAV network. The module senses communication signal frequencies from neighboring nodes and uploads them to the computer, while also broadcasting its own communication signals. MAC algorithms run independently on each computer. The computer is responsible for adjusting the frequency of the module’s next broadcast and calculating the average frequency based on the frequency uploaded by the module. Lastly, the wireless channel emulator, based on the experimental scenario selected by the console, sets the channel coefficients between all communication terminal pairs to simulate the actual scenario.

The scenario for this experiment is set as follows. We set up $N_{c}=30$, $c_{i}=520+3\left(i-1\right)$ MHz, and W=3 MHz. We run 50 sets of experiments, each with a randomly generated connected six-UAV network, with each node selecting a random frequency in the spectral range as the initial frequency. Because the number of time slots required by the algorithm is determined by the network topology, comparing the number of time slots becomes meaningless. We use the percent of the center frequency (PCF) as a performance parameter. It means the deviation percent of the final average frequency calculated by each node from the central frequency of the channel that the actual average frequency belongs to. Let ${\bar{F}}_{i}$ be the final frequency calculated for node *i*, $\bar{F}$ be the actual average frequency, and $c_{\bar{F}}$ be the channel that $\bar{F}$ belongs to. The performance parameter is shown in (9):

(9) $$PCF=\left({\bar{F}}_{i}-c_{\bar{F}}\right)/W$$

**Figure 9:**
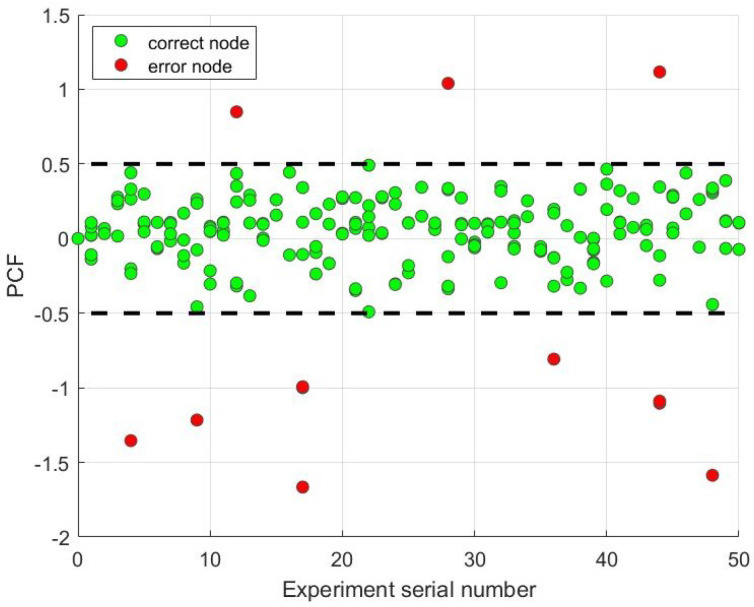
PCF values for each node in the physical experiment: PCF is the percent off the center frequency, which denotes the deviation of the frequency calculated by each node from the actual average frequency belongs to.

The results of our experiments are shown in Figure 9, where the vertical coordinate represents the final PCF values calculated by each node and the horizontal coordinates represent the experiment serial numbers. The green dots in the graph indicate that the node’s average frequency value is correct, while the red dots indicate that the node’s average frequency value is incorrect.

As shown in Figure 9, the average frequency values for a total of 600 nodes in 50 groups were incorrectly calculated for only 11 nodes, resulting in a correct rate of 98.17%, with 8 of the experiments having incorrectly calculated average frequency values and an 84% success rate for complete reconnection. This result demonstrates that the MAC algorithm can be applied to physical objects successfully.

### 5.2. Cause Analysis of Rendezvous Failures

There is an error in a real physical environment between the frequency value broadcast by the neighboring node and the frequency value sensed by the node. Typically, this error is negligible for a channel bandwidth of 3 MHz, as it is no more than 30 kHz. However, there is a special case where two neighboring nodes broadcast frequency values that are close to each other. For example, if one node broadcasts at 600 MHz and the other at 602 MHz, the node sensing the frequency cannot accurately distinguish between the two frequencies. Consequently, it can only sense the signals broadcast by the two neighboring nodes with the same frequency value, ranging between 600 MHz and 602 MHz. This results in a significant error between the sensing frequency value and the actual broadcast frequency value. Furthermore, as the algorithm iterates, the frequency values broadcast by the nodes converge to the average frequency value, thereby increasing the likelihood of this specific case.

Furthermore, a large number of iterations of the algorithm can result in computational errors. The number of iterations depends on the adjacency matrix, which has been analyzed in Section 4. Choosing a network with a larger diameter and average degree will result in a higher number of iterations. In addition, the computational accuracy also influences the number of algorithms in this physical experiment. The number of algorithm iterations is obtained from Algorithm 2, i.e., the number of iterations required to compute the rank of $F_{i}\left(t\right)$ and $F_{i}\left(t\right)|e$ when they are equal. To compute the rank of the matrix in the physical experiments, we use the Gaussian elimination method. We only require that the average frequency value calculated by the node at the end is within a certain range (PCF<0.5) for the channel intersection problem. Thus, it is important to choose a suitable calculation accuracy.

In conclusion, sensing frequency errors and a large number of iterations are the main causes of node calculation errors and rendezvous failures. Choosing networks with small diameters and averages, as well as suitable calculation accuracy, can reduce the likelihood of node calculation errors.

## 6. Conclusions

This paper introduces two algorithms, namely the MAC algorithm and the MRAC algorithm, for achieving channel rendezvous in multi-UAV networks. Each algorithm is tailored for specific scenarios. The MAC algorithm is designed for situations where UAVs can access the network adjacency matrix, and its algorithm efficiency is strongly correlated with the diameter and density of the UAV network. On the other hand, the MRAC algorithm is suitable for scenarios in which UAVs are equipped with multiple radios but cannot access the network adjacency matrix, and its efficiency increases with the number of radios. Simulation results demonstrate that both the MAC and MRAC algorithms outperform existing state-of-the-art algorithms in networks with different numbers of UAVs. Finally, this paper illustrates the effectiveness of the MAC algorithm through semi-realistic platform simulations, achieving an 84% reconnection success rate in 50 simulation experiments.

## Figures and Tables

**Table 1:** Parameter list.

Parameter	Variable
Number of UAVs	*m*
Number available	$N_{c}$
Distance between UAV *i* and UAV *j*	$D_{ij}$
Buffer distance	Δr
Sensing range	$R_{s}$
Adjacency matrix	A
Time to rendezvous	TTR
Vector of available channel	*C*
Vector of channel states	f
Number of subnets	$N_{s}$
Neighbors of UAV *i*	$Neigh_{i}$
Bandwidth of channel	*W*

## Data Availability

Due to confidentiality requirements, research data cannot be provided.

## References

[1] Shakeri R., Al-Garadi M.A., Badawy A., Mohamed A., Khattab T., Al-Ali A.K., Harras K.A., Guizani M. (2019). Design Challenges of Multi-UAV Systems in Cyber-Physical Applications: A Comprehensive Survey and Future Directions. IEEE Commun. Surv. Tutor..

[2] Liu Y., Dai H.N., Wang Q., Shukla M.K., Imran M. (2020). Unmanned Aerial Vehicle for Internet of Everything: Opportunities and Challenges. Comput. Commun..

[3] Li Y., Zhang H., Long K., Choi S., Nallanathan A. (2019). Resource Allocation for Optimizing Energy Efficiency in NOMA-based Fog UAV Wireless Networks. IEEE Netw..

[4] Xiong F., Li A., Wang H., Tang L. (2019). An SDN-MQTT Based Communication System for Battlefield UAV Swarms. Commun. Mag. IEEE.

[5] Koushik A.M., Hu F., Kumar S. (2019). Deep *Q* -Learning-Based Node Positioning for Throughput-Optimal Communications in Dynamic UAV Swarm Network. IEEE Trans. Cogn. Commun. Netw..

[6] Sodagari S., Jafarkhani H., Yousefi’Zadeh H. (2019). Improved Cognitive Radio Receivers Using Timing Mismatch of Primary and Secondary Users. Circuits Syst. II Express Briefs IEEE Trans..

[7] Theis N.C., Thomas R.W., Dasilva L.A. (2010). Rendezvous for Cognitive Radios. IEEE Trans. Mob. Comput..

[8] Yang B., Liu M., Li Z. (2018). Rendezvous on the Fly: Efficient Neighbor Discovery for Autonomous UAVs. IEEE J. Sel. Areas Commun..

[9] Guerra E.O., Reguera V.A., Duran-Faundez C., Nguyen T.M.T. (2022). Channel hopping for blind rendezvous in cognitive radio networks: A review. Comput. Commun..

[10] Wang J.H., Lu P.E., Chang C.S., Lee D.S. A Reinforcement Learning Approach for the Multichannel Rendezvous Problem. Proceedings of the 2019 IEEE Globecom Workshops (GC Wkshps).

[11] Yang B., Wei L., Zheng M., Liang Y.C. (2016). Fully Distributed Channel-Hopping Algorithms for Rendezvous Setup in Cognitive Multiradio Networks. IEEE Trans. Veh. Technol..

[12] Li J., Zhao H., Wei J., Ma D., Zhou L. (2016). Sender-Jump Receiver-Wait: A Simple Blind Rendezvous Algorithm for Distributed Cognitive Radio Networks. IEEE Trans. Mob. Comput..

[13] Watson C.L., Chakravarthy V.D., Biswas S. A multi-agent Q-learning based rendezvous strategy for cognitive radios. Proceedings of the Cognitive Communications for Aerospace Applications Workshop.

[14] Xiao L., Boyd S. Fast linear iterations for distributed averaging. Proceedings of the 42nd IEEE International Conference on Decision and Control (IEEE Cat. No.03CH37475).

[15] Chai Y., Cao K.C. Distributed UAV formation control with two-hop relay protocol. Proceedings of the 2017 36th Chinese Control Conference (CCC).

[16] Pasolini G., Dardari D., Kieffer M. (2020). Exploiting the Agent’s Memory in Asymptotic and Finite-Time Consensus Over Multi-Agent Networks. IEEE Trans. Signal Inf. Process. Netw..

[17] Mustafa A., Ul Islam M.N., Ahmed S., Tufail M.A. (2018). A Novel Approach for Fast Average Consensus under Unreliable Communication in Distributed Multi Agent Networks. Wirel. Pers. Commun..

[18] Lo F.W., Yang G.C., Chang M.K., Kwong W.C. (2023). An Improved Two-Hop Rendezvous Model with Buffer-Aided Relays in Channel-Hopping Cognitive-Radio Wireless Networks for Internet-of-Things. IEEE Internet Things J..

[19] Wang X., Li J., Xing J., Wang R., Xie L., Zhang X. (2016). A novel finite-time average consensus protocol for multi-agent systems with switching topology. Trans. Inst. Meas. Control.

[20] Hanada K., Wada T., Masubuchi I., Asai T., Fujisaki Y. (2021). Multi-agent consensus for distributed power dispatch with load balancing. Asian J. Control.

[21] Manitara N.E., Hadjicostis C.N. (2017). Distributed Stopping for Average Consensus in Digraphs. IEEE Trans. Control Netw. Syst..

[22] Xu P., Tian Z., Wang Y. An energy-efficient distributed average consensus scheme via infrequent communication. Proceedings of the 2018 IEEE Global Conference on Signal and Information Processing (GlobalSIP).

[23] Shi X., Bo B., Zhang Q., Chao S., Lv J. Consensus-Based Multi-UAV Target Tracking with Communication Delays. Proceedings of the 2017 9th International Conference on Intelligent Human–Machine Systems and Cybernetics (IHMSC).

[24] Khan U., Aeron S. (2016). Distributed subspace consensus in the presence of dynamic in-network disturbance. IEEE Trans. Control Netw. Syst..

[25] Li H., Zhang H., Wang Z., Zhu Y., Han Q. (2019). Distributed Consensus-Based Multi-Agent Convex Optimization via Gradient Tracking Technique. J. Frankl. Inst..

[26] Heinzelman W.R. Adaptive Protocols for Information Dissemination in Wireless Sensor Networks. Proceedings of the 5th Annual ACM/IEEE International Conference on Mobile Computing and Networking (MobiCom’99).

[27] Stevenson C., Chouinard G., Lei Z., Hu W., Shellhammer S., Caldwell W. (2009). IEEE 802.22: The first cognitive radio wireless regional area network standard. IEEE Commun. Mag..

[28] Gu Z., Wang Y., Shen T., Lau F. (2020). On Heterogeneous Sensing Capability for Distributed Rendezvous in Cognitive Radio Networks. IEEE Trans. Mob. Comput..

[29] Han H., Gao W., Yan H., Bai J., Tang Y. Networked Robotic System Simulation Platform: Hardware-in-the-Loop Architecture, Enabling Technologies and Implementations. Proceedings of the 2020 International Conference on Wireless Communications and Signal Processing (WCSP).

